# JAK inhibitors: an evidence-based choice of the most appropriate molecule

**DOI:** 10.3389/fphar.2024.1494901

**Published:** 2024-10-29

**Authors:** Luca Antonioli, Alessandro Armuzzi, Massimo C. Fantini, Matteo Fornai

**Affiliations:** ^1^ Unit of Pharmacology and Pharmacovigilance, Department of Clinical and Experimental Medicine, University of Pisa, Pisa, Italy; ^2^ IBD Center, IRCCS Humanitas Research Hospital, Rozzano, Italy; ^3^ Department of Biomedical Sciences, Humanitas University, Pieve Emanuele, Italy; ^4^ Department of Medical Sciences and Public Health, University of Cagliari, Cagliari, Italy; ^5^ Gastroenterology Unit, Azienda Ospedaliero Universitaria di Cagliari, Cagliari, Italy

**Keywords:** JAK inhibitors, pharmacokinetic, pharmacodynamic, efficacy, safety

## Abstract

Janus kinase inhibitors (JAKis) represent a fundamental therapeutic tool for the treatment of patients with immune-mediated inflammatory diseases. Although JAKis are often considered a homogeneous class of drugs whose members are thought to be largely interchangeable, there are significant differences in their efficacy and safety profiles. This narrative review analyzes the pharmacokinetic and pharmacodynamic differences among JAKIs, highlighting their clinical relevance based on the most recent available evidence. The article aims to provide rheumatologists, gastroenterologists and dermatologists with practical guidance for choosing the most appropriate JAKi for each patient, given the lack of evidence-based recommendations on this topic, to improve clinical outcomes. Due to its preferential action on JAK1, intestinal metabolization and proven absence of impact on male fertility, filgotinib may be characterized by an improved benefit/risk ratio compared with other less selective JAKis.

## 1 Introduction

Immune-mediated inflammatory diseases (IMIDs) are a heterogeneous group of chronic conditions that share common pathways (Sozzani et al., 2014). The most commonly affected body sites are the joints, skin and gastrointestinal system, giving rise to diseases such as rheumatoid arthritis (RA), juvenile idiopathic arthritis (JiA), psoriatic arthritis (PsA), axial spondyloarthritis (axSpA), atopic dermatitis (AD), alopecia areata (AA) and inflammatory bowel disease (IBD) (Sozzani et al., 2014; Schwartz et al., 2017).

IMIDs are among the most common diseases in Western countries (Sozzani et al., 2014) and exert a significant impact on patients, not only because they can lead to the destruction of affected tissues but also because of their adverse effects on health-related quality of life, daily activities and social functioning (Tran et al., 2021).

Although IMIDs are substantially heterogeneous from a pathophysiologic standpoint, they are all characterized by a persistent and over production of proinflammatory cytokines caused by a dysregulation of the immune system (Lai and Dong, 2016).

According to the most recent evidence, the IMIDs classification is currently transitioning from being organ-based to be molecular-based, where each condition can be is defined by a signature cytokine hub (Schett et al., 2021). In RA, for instance, interleukin (IL)-6 represents a critical pathogenetic node, while IL-23 and IL-17A are the key drivers of intestinal and spinal inflammation in IBD and axSpA respectively. In this model, TNF-α probably represents a shared pathway acting downstream in the inflammatory process of all the IMIDs.

Cytokines and other molecules involved in the inflammatory process have become an important therapeutic targets for the treatment of IMIDs and the development of neutralizing monoclonal antibodies and recombinant proteins against these targets has revolutionized the treatment of these conditions (Schwartz et al., 2017).

Recently, Janus kinase inhibitors (JAKi) have been developed to block the effect of proinflammatory cytokines in IMIDs. These small molecule drugs do not bind directly to specific cytokines but act downstream in the inflammatory cascade: they block the JAK/STAT pathway, which is essential for the intracellular signal transduction triggered by cytokines binding to their receptors on cell membranes (Xin et al., 2020).

Tofacitinib was the first available JAKi, approved in 2012 by the Food and Drug Administration (FDA) and in 2017 by the European Medicines Agency (EMA) for the treatment of RA; currently, four JAKi are available in the European Community (EC) for the treatment of RA, namely baricitinib, filgotinib, upadacitinib and tofacitinib (Wlassits et al., 2024; European Medicine Agency, 2024d).

Filgotinib, the most recent molecule in the JAKi class, was approved on 20 September 2020 in the European Union and Japan; it was developed to be an ATP-competitive, reversible, JAK1 preferential inhibitor, for the treatment of inflammatory disease such as RA and ulcerative colitis (UC) (Dhillon and Keam, 2020).

After more than a decade of clinical use, JAKis are now recognized as an important therapeutic tool for treating patients affected by IMIDs, owing to their efficacy and the convenience of oral administration (Wlassits et al., 2024).

However, although all JAKis share the capacity to inhibit the activity of JAK proteins, significant differences in the selectivity towards the different JAK family members (i.e. JAK1, JAK2, JAK3 and tyrosine kinase 2 [TYK2]) exist, with important implications in terms of efficacy and safety profile. In spite of these differences, these drugs are still considered interchangeable and there are no evidence-based recommendations to guide clinicians in choosing the most appropriate JAKi based on each patient’s individual characteristics.

This paper aims to clearly define the differences between the four JAKis available in EC for the treatment of RA based on their pharmacodynamics/pharmacokinetic properties and then to discuss how these differences may impact on the clinical use of these drugs. In addition, it provides general indications for the selection of patients to be treated with different molecules.

## 2 Pharmacodynamic considerations

### 2.1 Cytokine signaling through the JAK/STAT system and its inhibition

Cytokines are a heterogeneous group of proteins that exert critical biological effects, including the modulation of acute and chronic inflammatory processes (Turner et al., 2014). It is widely acknowledged that these molecules’ dysregulation plays a crucial role in IMIDs pathogenesis since the overexpression of proinflammatory cytokines is a hallmark of these conditions (Schwartz et al., 2017).

The action of cytokines is mediated by binding to different types of receptors (Schwartz et al., 2017), which, from a biochemical point of view, are all transmembrane glycoproteins composed of different subunits (Bagley et al., 1997). Cytokine receptors are classified into several families, including the tumor necrosis factor (TNF) family receptors, the transforming growth factor receptor superfamily, the tyrosine kinase receptor superfamily, the G protein-coupled receptor superfamily, and the Type 1 and Type 2 receptor superfamily (Schwartz et al., 2017; Turner et al., 2014). It has been shown that the pathogenesis of IMIDs predominantly involves proinflammatory cytokines acting on Type 1 and Type 2 receptors (Banerjee et al., 2017). Notably all Type 1 and Type 2 receptors use the Janus kinase/Signal Transducer and Activator of Transcription (JAK/STAT) for signal transduction (Banerjee et al., 2017), which therefore represents a target for disrupting proinflammatory cytokines signalling in IMIDs.

JAKs are a key component of the JAK/STAT system: they are kinases associated with the intracellular domain of Type 1 and 2 receptors and exist in four different isoforms: JAK1, JAK2, JAK3 and TYK2 (Schwartz et al., 2017). For cytokine signal transduction, at least two JAKs must be present in the receptor, either of the same type or two different types (Choy, 2019).

**FIGURE 1:**
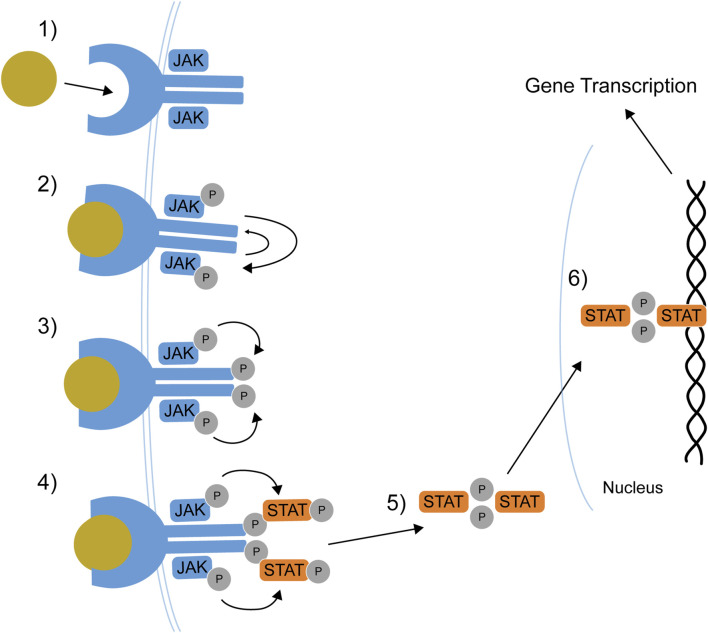
The JAK-STAT pathway. 1) Cytokine binds its receptor. 2) Receptor-associated JAKs transphosphorylate and activate each other. 3) JAKs phosphorylate the receptor tail. 4) STATs dock on receptor tail and are phosphorylated. 5) STATs dissociate from the receptor and dimerizes. 6) STAT dimers translocate to the nucleus where they regulate gene transcription. JAK: Janus kinase, P: phosphate group, STAT: Signal Transducer and Activator of Transcription. Adapted from Lin et al. (2020).

Cytokines’ signal transduction starts when a cytokine binds to its receptor; this bond drives conformational changes in the receptor and results in the phosphorylation of specific tyrosine residues on JAKs by ATP molecules (Figure 1) (O’Shea et al., 2015). JAK phosphorylation results in phosphorylation and dimerization of STAT proteins in the cytoplasm; once dimerized, STATs translocate into the nucleus, where they activate the transcription of gene coding for cytokine production. This ultimately determines production and release in the extracellular milieu, thus determining the biological activity of cytokines (Schwartz et al., 2017; Banerjee et al., 2017).

JAKis block the ATP binding on JAKs, impeding the phosphorylation of these proteins and the subsequent cascade of events leading to cytokine signal transduction (Lin et al., 2020).

### 2.2 Different JAKs, different biological effects

**FIGURE 2:**
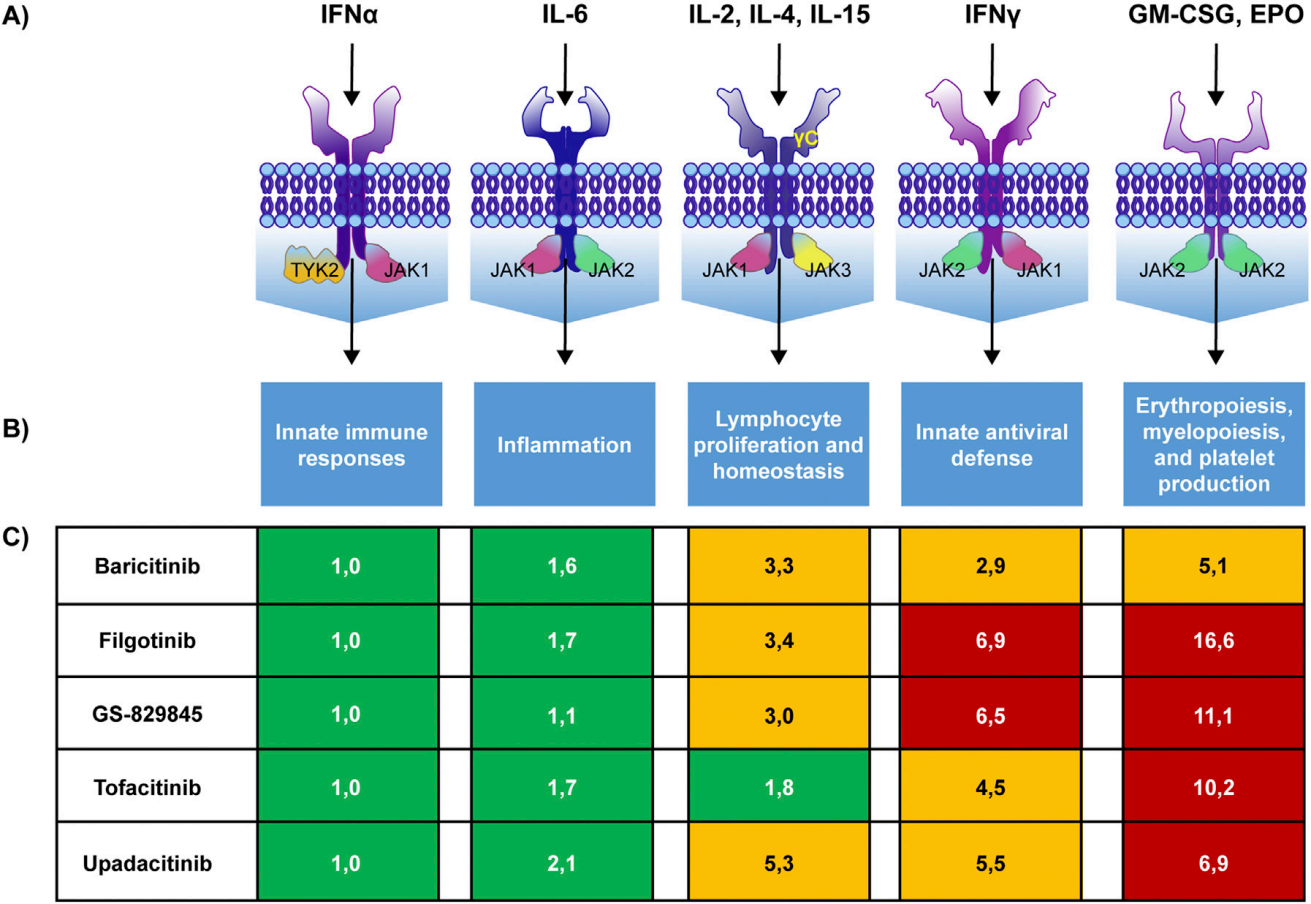
JAK pairing patterns, their biologic effects, and JAKis selectivity. JAK pairing patterns associated with cytokine receptors **(A)**, biological effects of the pathway **(B)** and selectivity of different JAKis for each pathway **(C)** with higher numbers denoting lower selectivity. GS-829845 is the filgotinib active metabolite. Colours indicate the fold selectivity compared with JAK1/TYK2 pathway inhibition. Green: 1–2.5 fold reduction. Yellow: >2.5–6 fold reduction. Red: >6 fold reduction. IFN: interferon, GM-CSF: Granulocyte-Macrophage Colony-Stimulating Factor, EPO: erythropoietin. Adapted from Traves et al., 2021.

Different cytokine receptors use distinct combinations of JAK isoforms for signal transduction (Figure 2) (Traves et al., 2021). This is an essential factor to consider when designing or choosing a JAKi since the different selectivity for JAK isoforms may lead to different biological effects, influencing the efficacy and safety profile of a drug (Lin et al., 2020; O’Shea et al., 2015; Traves et al., 2021). Based on the current available evidence, among the different JAK isoforms, only JAK1 and TYK2 seem to be involved almost exclusively in inflammatory signal transduction and may represent an optimal target for controlling disease activity in IMIDs (Traves et al., 2021). Several studies suggest that the efficacy of JAKis in RA relies mainly on JAK1 inhibition (Cox and Cools, 2011; Haan et al., 2011). Similarly, the most critical proinflammatory cytokines involved in the pathogenesis of IBD seem mediate their effect via JAK1 and TYK2 (Harris and Cummings, 2021).

In contrast, JAK2 and JAK3 also regulate other essential physiological processes (Traves et al., 2021).

For instance, a pre-clinical study, in which a conditional knockout approach was used to inactivate JAK2 at any stage of prenatal or postnatal development, showed that JAK2 plays a key and non-redundant role in hematopoiesis. Adult mice in which JAK2 had been inactivated showed a reduction in blood cell counts, abnormal erythrocyte morphology, reduction of bone marrow hematopoietic potential, and splenic atrophy (Park et al., 2013).

This evidence is not surprising, considering that JAK2 is present in the form of a homodimer on the erythropoietin and Granulocyte-Macrophage Colony-Stimulating Factor (GM-CSF) receptor, playing a crucial role in erythropoiesis, myelopoiesis and platelet production. (Traves et al., 2021).

JAK2 is also associated with the myeloproliferative leukaemia (MPL) receptor, which is stimulated by thrombopoietin (TPO) and is crucial for platelet production. An *in vitro* study has shown that, in the presence of TPO, exposure to suboptimal doses of a JAK2 inhibitor leads to a paradoxical increase in platelet production both *in vitro* (in CD34^+^ cells) and *in vivo* (in C57BL/6 mice) (Besancenot et al., 2014).

In addition, the JAK2 homodimer is associated with leptin receptors and transduces the leptin signal of this adipokine by phosphorylating STAT3. Leptin is an adipokine that regulates energy homeostasis and glucose and lipid metabolism. It has been shown that hyperphagia and obesity occur in cases of congenital leptin deficiency or loss-of-function mutations of this adipokine receptor (Park and Ahima, 2014).

Finally, JAK3 is associated with JAK1 in the IL-15 receptor, representing the dominant JAK for signal transduction. IL-15 receptor has been shown to be critical for the development and functioning of natural killer (NK) cells. These immune cells play a crucial role in defending the body against viruses and cancer (Schwartz et al., 2017).

It should be emphasized, however, that the whole picture of the biological effects associated with each JAK isoform and the role of this kinase’s different variants in IMIDs pathophysiology are not yet fully clarified; therefore, what is stated above in this section should be considered only as a selection of the most accepted available evidence.

### 2.3 Assessment of the preferential selectivity of JAKis

JAK family members are structurally homologous and share a highly conserved ATP binding pockets. JAKis compete with ATP for binding these pockets and differences in affinity for the pockets of each single JAK result in the distinct affinity profiles of these drugs for JAK members (Lin et al., 2020).

The potency of JAKis in the inhibition of the different JAKs isoforms was first evaluated *in vitro* by Dowty et al. with enzymatic assay (Table 1). According to the IC_50_ values obtained with this method, all JAKis inhibit JAK1 with greater potency compared with other JAKs, and significant differences exist in the inhibitory profile of different drugs (Dowty et al., 2019). However, this type of assay has significant limitations. For instance, it does not provide information regarding the effect of JAKis on the JAK complexes found in biological systems. Moreover, it cannot show the impact of JAK inhibition on receptor signal transduction.

**TABLE 1:** Mean IC_50_ values obtained in enzymatic assay for tofacitinib, baricitinib, upadacitinib, and filgotinib inhibition of JAK1, JAK2, JAK3, and TYK2.

	IC_50_ (nmol/L)			
	JAK1	JAK2	JAK3	TYK3
Tofacitinib	15	71	45	472
Baricitinib	0.78	2	253	14
Upadacitinib	0.76	19	224	118
Filgotinib	45	357	9,097	397

IC_50_ values represent the geometric mean of independent experiments carried out in the presence of 1 mmol/L ATP. IC_50_: half-maximum inhibitory concentration, JAK: janus kinase, ATP: Adenosine TriPhosphate. Adapted from Dowty et al. (2019).

To overcome these limitations, an *in vitro* study was carried out using a model based on peripheral blood mononuclear cells (PBMCs) and whole blood (WB) taken from healthy volunteers and RA patients. The ability of tofacitinib, baricitinib, upadacitinib and filgotinib to inhibit signal transduction (percentage of STAT phosphorylation inhibition) in these cells, in different JAK/STAT pathways, following cytokine stimulation was assessed with flow cytometry (Traves et al., 2021).

The study showed that all JAKis block the IFN-alpha and IL-6 signaling pathways, both of which are dependent on JAK1, with no significant differences between drugs.

On the contrary, there were significant differences in the effect of JAKis on the other pathways. Importantly, filgotinib showed the lowest inhibitory potency on the IFN-gamma (JAK1/JAK2), IL-2/IL-15/IL-16 (JAK1/JAK3), G-CSF/IL-12/IL-23 (JAK2/TYK2) and GM-CSF (JAK2/JAK2) pathways compared with all other drugs in the class (Figure 2).

In general, the *in vitro* studies carried out so far show that tofacitinib can be considered a pan-inhibitor, since it inhibits all JAK isoforms indiscriminately; baricitinib, on the other hand, has prevalent selectivity for JAK1 and JAK2; upadacitinib and especially filgotinib are JAK1 preferential inhibitors, with limited effects on other isoforms (Liu et al., 2021).

### 2.4 From pharmacodynamics to clinical safety

Adverse events such as infections, venous thromboembolism (VTE) events, cancer, and blood cell cytopenia are considered class effects of JAKis by regulatory agencies. However, differences observed in the pharmacodynamic profiles of JAKis along with the results from pivotal trials weaken the strength of this assumption.

Currently is well established that JAKis’ efficacy is associated with their preferential selectivity for JAK1, while safety concerns emerge as inhibition of JAK2- and JAK3-dependent pathways increases (Traves et al., 2021).

Therefore, the reduced inhibition of JAK2 and JAK3-dependent cytokine signaling pathways by filgotinib may theoretically explain its improved tolerability profile, as observed in RA phase 3 studies (Table 2).

**TABLE 2:** Efficacy and safety finding from phase 3 studies of JAKis in RA.

	Filgotinib	Tofacitinib	Upadacitinib	Baricitinib	
	200 mg	5 mg bid	15 mg	2 mg	4 mg
MTX-IR					
ACR20	19%	25%	31%	NR	37%
ACR50	25%	26%	33%	NR	32%
ACR70	21%	15%	25%	NR	22%
DAS28-hsCRP ≤3.2	32%	5%	32%	NR	33%
Biologic-IR					
ACR20	35%	17%	37%	22%	28%
ACR50	28%	18%	22%	12%	20%
ACR70	15%	12%	5%	11%	9%
DAS28-hsCRP ≤3.2	14%	4%	20%	9%	8%
Haemoglobin (g/dL)	+0.2	+0.08	−0.8	−0.28	−0.2
HZ	0.1% (0.3% placebo)	1%–10%	0.7%	1.4%	4.3%
Infections (E/100PY)	26.5	43.9	93.7	101	
Serious infections (E/100PY)	1.7	2.4	3.8	3.2	
Opportunistic infections (E/100PY)	0.1	<0.1	0.6	0	0.5
VTE (E/100PY)	0.2	0.27	0.5	0.6	0.8

MTX-IR: methotrexate inadequate response patients, ACR: american college of rheumatology, DAS28-hsCRP: Disease Activity Score for 28-joint count with serum high-sensitivity C-reactive protein, Biologic-IR: biologic inadequate response patients, HZ: herpes zoster, E/100PY: events per 100 patient-years, RA: rheumatoid arthritis, VTE: venous thromboembolism. Adapted from Traves et al. (2021).

Although a direct comparison of different JAKis’ safety profiles is not available and even though the rates of AEs are low, in these studies filgotinib, compared with tofacitinib, upadacitinib and baricitinib, showed numerically lower incidences of infections (26.5, 43.8, 93.7 and 101 events/100 patient-years, respectively) and serious infections (1.7, 2.4, 3.8 and 3.2 events/100 patient-years, respectively). Similar results were obtained for herpes zoster events, whose frequency was numerically lower with filgotinib compared with the other JAKis (0.1% vs. 1%–10% with tofacitinib, 0.7% with upadacitinib and 1.4%–4.3% with baricitinib) (Traves et al., 2021).

It is not feasible to indirectly compare JAKis safety profiles in IBDs, since the studies in this setting show large differences in design and patients’ clinical/demographic characteristics (Feagan et al., 2021a; Danese et al., 2022; Loftus et al., 2023; Sandborn et al., 2017). However, the differences in infection rates in favour of filgotinib compared with the other JAKis observed in RA are likely confirmed in IBDs since the safety profile of JAKis in these indications is consistent with that observed in RA (SmPC filgotinib; SmPC upadacitinib; SmPC tofacitinib). Moreover, the filgotinib safety profile in UC is well-known and reassuring, as shown by the results of the SELECTION study, a randomized, placebo-controlled, double-blind, phase 3 trial in nearly 660 patients (Feagan et al., 2021a). In SELECTION, rates of serious adverse events and discontinuations due to adverse events were similar in the filgotinib and placebo arms. In the maintenance phase up to 58 weeks, herpes zoster infections and serious infections rates were low with filgotinib (0.5% and 1.0%–1.7%, respectively) and similar to those observed in placebo groups (1.1% and 1.1%–2.2%, respectively). Notably, these low rates of infection were reported in the SELECTION trial despite the concomitant use of corticosteroids and the fact that immunosuppressants were permitted, by contrast with the tofacitinib phase 3 trial in which these treatments were discontinued before initiation of induction.

**FIGURE 3:**
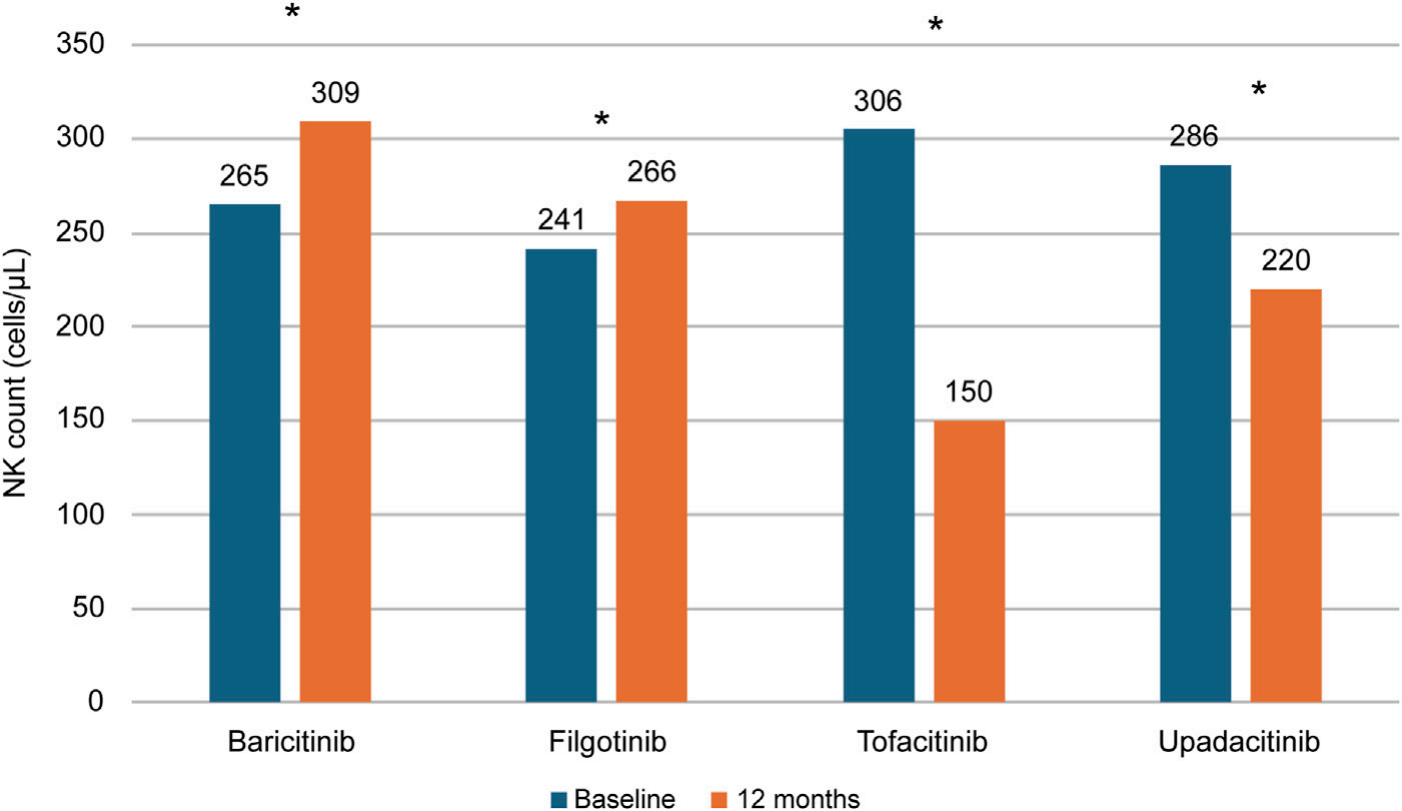
NK count changes in patients with RA treated with different JAKis. Flow cytometry-assessed NK counts in 115 RA patients treated with baricitinib, filgotinib, tofacitinib or upadacitinib in the Italian real-world study ELECTRA-i. *P = 0.0001 baseline vs. 12 months. Adapted from Benucci et al. (2024). NK: Natural Killer. RA: Rheumatoid Arthritis.

Based on these observations, it is tempting to speculate that filgotinib, having a lower inhibitory effect on JAK3 as previously mentioned, does not impact the development and maturation of NK cells, immune cells involved in the defence against infections. This hypothesis is supported by *in vitro* data and in a recent observational study (Di Paolo et al., 2019; Benucci et al., 2024). *In vitro*, filgotinib has been shown to exert a reduced inhibitory effect on IL-15-induced NK proliferation compared with tofacitinib, baricitinib and upadacitinib (Di Paolo et al., 2019). In the Italian real-world study ELECTRA-i, carried out on 115 patients with RA, after 12 months of treatment with filgotinib and baricitinib the blood counts of NK CD56^+^ counts increased in a statistically significant fashion, since these drugs are not able to bind JAK3 (Figure 3). Interestingly, the NK cell levels were significantly reduced with tofacitinib and upadacitinib, the two JAKis that also inhibit JAK3 (Benucci et al., 2024).

Another consequence of JAKis selectivity differences is the impact of these drugs on haemoglobin levels. In phase 3 clinical trials, a reduction in haemoglobin levels ranging from −0.2 to −2.8 g/dL was observed in patients treated with upadacitinib and baricitinib, whereas the levels with tofacitinib remained broadly stable (Table 2) (Traves et al., 2021). In contrast, an increase of 0.2 g/dL in haemoglobin levels was observed in patients treated with filgotinib. These results might be attributed in part to the reduced affinity of filgotinib for JAK2 which, as we have seen, is the JAK member involved in the signal transduction of EPO and GM-CSF. That filgotinib does not affect hematopoietic processes is further supported by the absence of clinically relevant changes in leukocyte and platelet counts reported in phase 3 clinical trials (European Medicine Agency, 2024a).

### 2.5 ORAL surveillance study results in the light of JAKis pharmacodynamics

ORAL Surveillance was an FDA-mandated, post-marketing, non-inferiority trial conducted to collect additional safety data about tofacitinib in RA patients. This trial was conducted as a result of concerns raised by an increased risk of cancer, cardiovascular events, and serious infections observed in the tofacitinib developmental program with the higher, unapproved dose of 10 mg twice daily (Winthrop and Cohen, 2022).

The study showed a dose-dependent higher risk of cancer, Major Adverse Cardiovascular Events (MACE), infections and VTE in patients treated with tofacitinib compared with TNF inhibitors (Ytterberg et al., 2022).

The ORAL Surveillance study results have led international regulatory authorities such as the FDA and the European Medicines Agency (EMA) to restrict the use of all the JAKis in elderly patients (≥65 years) and in those with cardiovascular or malignancy risk factors, irrespective of the therapeutic indication (Winthrop and Cohen, 2022).

This decision appears to be based on a prudential approach, and it is certainly valuable for the sake of patient protection. However, the increase in knowledge about the pharmacodynamic differences between JAKis, combined with a growing amount of reassuring real-life safety evidence, leads to question the correctness of this extrapolation.

First of all, it should be remembered that in IMIDs such as RA and IBD the risk of infections and thromboembolic cardiovascular events is increased compared with the general population, regardless of the type of therapy, and it correlates with the severity of the inflammation (Fragoulis et al., 2020; Yerushalmy-Feler et al., 2018; Atzeni et al., 2017; Singh et al., 2015; Tilg et al., 2023). Therefore, the results of clinical trials evaluating the safety of treatments for IMIDs should be interpreted taking into account this epidemiological and pathophysiological evidence.

Moreover it should be emphasized that the ORAL Surveillance study provides results obtained in a very limited subset of patients who are candidates to receive JAKis, i.e., subjects with RA, age ≥50, and at least one cardiovascular risk factor. Moreover, it is now established that JAKis selectivity profile can potentially impact the propensity of each drug to cause the ORAL Surveillance safety endpoints.

It is therefore conceivable that the use of a JAKi with preferential selectivity for JAK1, which therefore has a predominantly anti-inflammatory action and does not inhibit kinases involved in response to infections and tumours (JAK3) or the production of platelets and other blood cells (JAK2) may be associated with a better safety profile than the pan-inhibitor tofacitinib.

**FIGURE 4:**
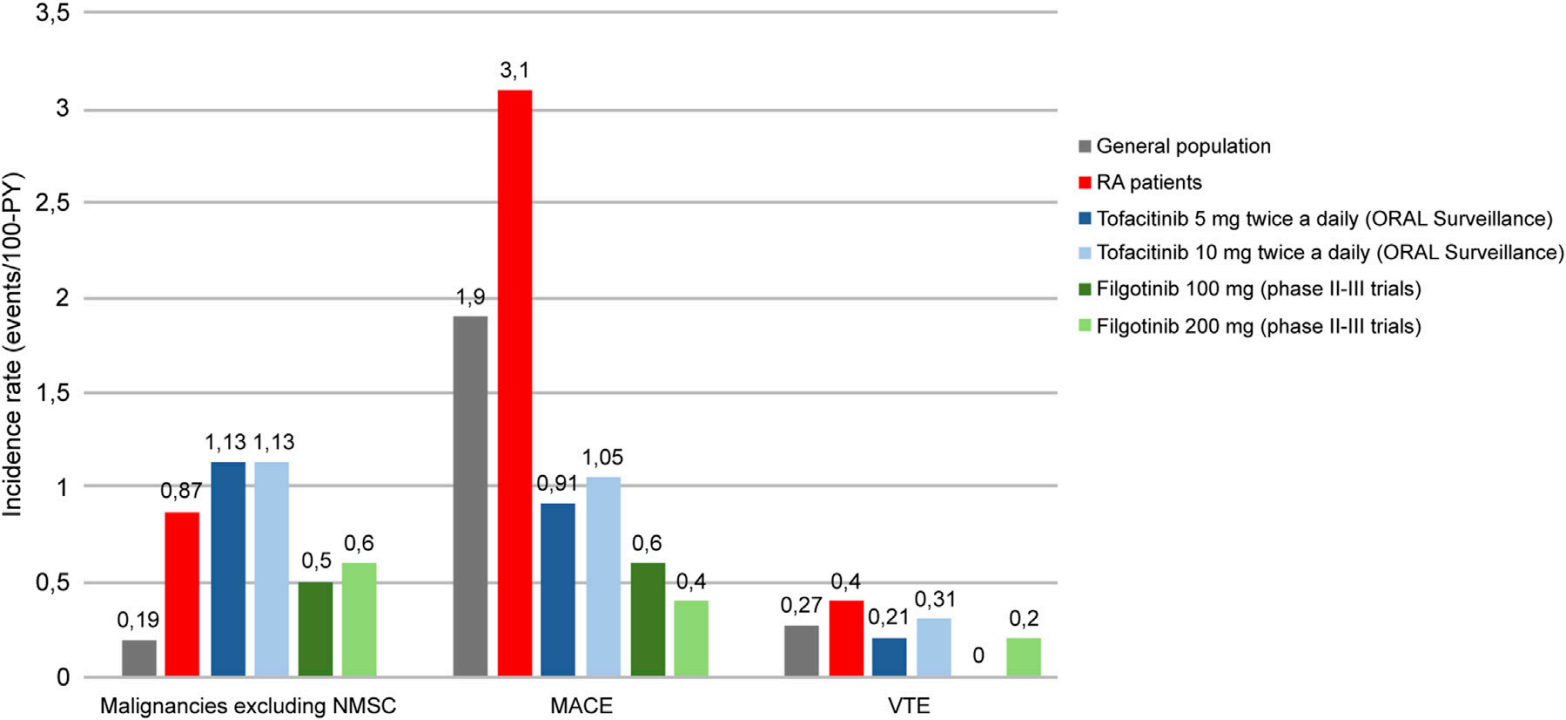
Incidence rates of malignancies excluding NMSC, MACE and VTE in general population, RA patients and RA patients treated with tofacitinib (5/10 mg twice daily) or filgotinib (100 mg/200 mg). RA: rheumatoid arthritis, PY: patient-years, NMSC: Non Melanoma Skin Cancer, MACE: Major Adverse Cardiovascular Event, VTE: Venous thromboembolism. Adapted from World Cancer Research Fund International (2024), Askling et al. (2016), Raadsen et al. (2021), Ketfi et al. (2021), Wendelboe and Raskob (2016), Winthrop et al. (2022), Ytterberg et al. (2022).

Safety results from the development program of filgotinib in RA corroborate this hypothesis. An integrated safety analysis of data from four phase III and three phase II filgotinib studies in RA, including 3,691 patients for 6,080.7 Patient-Years Exposition (PYE), showed that both doses of this JAKi (100 and 200 mg) were generally well tolerated (Winthrop and Cohen, 2022). Exposure-Adjusted Incidence Rates (EAIR) per 100 PYE of malignancies excluding Non Melanoma Skin Cancer (NMSK), MACE and VTE were low in the long-term filgotinib use (0.5–0.6, 0.4–0.6, and 0–0.2, respectively), with values numerically lower compared with ORAL Surveillance tofacitinib results (Figure 4) (Winthrop and Cohen, 2022; Ytterberg et al., 2022). The incidence of these adverse events is known to be higher in RA patients than in the general population; however, incidence rates reported during filgotinib treatment seem to be similar to those reported in subjects without RA (World Cancer Research Fund International, 2024; Askling et al., 2016; Raadsen et al., 2021; Ketfi et al., 2021; Wendelboe and Raskob, 2016; Winthrop and Cohen, 2022; Ytterberg et al., 2022).

## 3 Pharmacokinetic considerations

### 3.1 JAKis pharmacokinetic profiles

JAKis pharmacokinetic profiles are summarised in Table 3. All JAKis are characterised by T_max_ ranging from 0.5 to 4 h after oral intake, indicating a certain grade of variability in absorption rates between the drugs (European Medicine Agency, 2024d; European Medicine Agency, 2024b; European Medicine Agency, 2024c; European Medicine Agency, 2024a). Oral bioavailabilities, i.e. the percentages of the administered dose that reach the blood in an active form, are similar and relatively high (above 70%). Likewise, the percentages of drug transported in the bloodstream bound to proteins is around 50% for all JAKis.

**TABLE 3:** Summary of pharmacokinetic parameters of different JAKis.

	Tmax (h)	Oral bioavailability (%)	Vd (L)	Plasma protein binding (%)	Metabolism	Substrate for OATP1B1/1B3	T1/2 (h)	Excretion^a^
Tofacitinib	0.5–1	74	87	40%	CYP3A4 >CYP2C19	No	2.3–3.1	Urine (30%)
Baricitinib	0.5–3	80	76	50%	CYP3A4	No	8	Urine (75%) Feces (20%)
Upadacitinib	2–4	76^b^	294^b^	52%	CYP3A4 >CYP2D6	-	9–14	Urine (24%) Feces (38%)
Filgotinib	2–3	-	4.7	55–59	CES2 >CES1	Yes	7	Urine (9.4%) Feces (4.5%)
GS-829845	5	-	4.4	39–44	-	Yes	19	Urine (54%) Feces (8.9%)

Tmax: time to peak drug concentration, Vd: Volume of distribution, T1/2: terminal half-life, CES: carboxyl-esterase. Adapted from Dhillon and Keam (2020), European Medicine Agency (2024d), European Medicine Agency (2024b), European Medicine Agency (2024c), European Medicine Agency (2024a).

However, substantial differences exist in the elimination half-life (T1/2) of these drugs, ranging from 2.3–3.1 h for tofacitinib (which is, therefore, the only listed JAKi requiring to be administered twice daily) to 19 h for the active metabolite of filgotinib.

Notably, filgotinib is the only JAKi with an active metabolite (GS-829845). This molecule exhibits a similar selectivity for JAK1 compared with filgotinib but inhibits this kinase with a tenfold lower potency. The lower potency is partly compensated by GS-829845 high half-life and thus the metabolite makes a relevant contribution to the overall filgotinib pharmacodynamic effect (European Medicine Agency, 2024a).

Owing to its potential impact on clinical practice, the most relevant difference between JAKis is the metabolization pathway. All JAKis, except filgotinib, are biotransformed by hepatic enzyme systems, predominantly cytochrome P450 isoenzyme CYP3A4. Filgotinib, on the other hand, is metabolised in the gut, predominantly by carboxyl-esterase 2 (CES2) and, to a lesser extent, by carboxyl-esterase 1 (CES1).

Another difference in the pharmacokinetic profile of JAKi is their interaction with the hepatic uptake transporters Organic Anion Trasporting Peptide (OATP)-1B1 and -1B3. Only filgotinib and its metabolite are substrates of OATP-1B1 and OATP-1B3 and this could have clinically relevant effects when this JAKi is co-administered with other drugs using the same transporters, such as statins.

### 3.2 Impact of JAKis metabolization pathway in poly-treated patients

Two recent literature reviews (Veeravalli et al., 2020; Namour et al., 2022) summarised the possible pharmacokinetic interactions between JAKis and other drugs.

The first review evaluated tofacitinib, baricitinib, and upadacitinib. The administration of baricitinib was shown to be associated with a 30% reduction of simvastatin C_max_, while the administration of upadacitinib reduced rosuvastatin and atorvastatin C_max_ by approximately 20%. Therefore, in patients treated with these JAKis, statins efficacy could be lower than expected (Veeravalli et al., 2020). Not unexpectedly, the administration of fluconazole or ketoconazole (known inhibitors of many drugs’ metabolism) increases C_max_ of all the JAKis, with a possible higher risk of adverse events. In contrast, the administration of rifampicin (an inducer of liver enzymes and transporters) greatly reduces tofacitinib, baricitinib, and upadacitinib AUCs (Veeravalli et al., 2020).

The second review focused on filgotinib (Namour et al., 2022). Based on its intestinal metabolism, filgotinib does not exert a clinically relevant inhibitory or inducing effect on hepatic cytochromes, the enzymes most commonly involved in drug interactions. Therefore, filgotinib is characterized by a reduced potential for interaction with other drugs, and its use may be particularly appropriate for poly-treated patients using drugs with predominant hepatic metabolism (Dhillon and Keam, 2020). Rifampicin was the only drug capable of interacting with the active metabolite of filgotinib, reducing its AUC. Moreover, filgotinib showed a non-significant trend to increase rosuvastatin C_max_and AUC because these drugs use the same transport systems (Namour et al., 2022).

### 3.3 Effect of JAKis on lipid profile

A well-known JAKi class effect is the increase in blood cholesterol levels. In this regard, it is helpful to analyze the evidence regarding tofacitinib, since it was the first JAKi to be approved and hence also the one with the longest safety follow-up available.

Among the most comprehensive data on the impact of tofacitinib on lipid levels are those collected during the OCTAVE study program in which the JAKI was administered to over 1,100 patients with UC (Sandborn et al., 2017). The OCTAVE study program included two 8-week induction studies, followed by a 52-week maintenance study (OCTAVE Sustain) and an Open Label Extension (OLE) for a total drug exposure of about 7 years (Feagan et al., 2021b; Sandborn et al., 2022).

Tofacitinib altered the lipid profile of UC patients as early as the induction phase of the OCTAVE study, in which a slight increase in both LDL-c and HDL-c compared with placebo was observed (Sands et al., 2020). This effect was dose-dependent and reversible on drug withdrawal. Indeed, while in the maintenance phase, LDL-c and HDL-c levels remained consistently elevated in patients who continued treatment with tofacitinib, in those who switched to placebo these lipids levels quickly normalized. Interestingly, the LDL-c/HDL-c ratio remained stable during the maintenance phase of the OCTAVE study (Sands et al., 2020). HDL-c and LDL-c remained almost unchanged in OLE patients treated with the 10 mg BID dose of tofacitinib, while they decreased over time in those treated with the 5 mg BID dose (Sands et al., 2021).

In the OCTAVE study program, the highest tofacitinib-related increases in cholesterol levels occurred in patients with higher levels of this lipid at baseline (Sands et al., 2021).

Filgotinib and baricitinib showed similar effects on cholesterol levels to tofacitinib. Filgotinib impact on lipid profile has been evaluated in the SELECTION study (Feagan et al., 2021a). In this trial filgotinib resulted in minor increases in total cholesterol, LDL-c and HDL-c during the induction phase (10 weeks). These changes are not clinically relevant since in filgotinib highest dose group (200 mg), the cholesterol increases were similar to those observed in the placebo group (total cholesterol +29.3 mg/dL vs. +29.1 mg/dL respectively; LDL-c +24.2 mg/dL vs. +23.2 mg/dL; HDL-c +16.0 mg/dL vs. 11.9 mg/dL). Moreover, during the SELECTION study 52-week maintenance phase, cholesterol levels in both filgotinib arms remained stable.

Despite the increase in cholesterol being a class effect, significant differences between JAKis can be observed. A recent meta-analysis of patients with rheumatoid arthritis showed that filgotinib increased LDL-c and HDL-c levels to a similar extent while maintaining their ratio approximately constant (Li et al., 2022). In contrast, with tofacitinib and upadacitinib, LDL-c increases more than HDL-c. These differences between JAKis have also been confirmed in a recent Italian real-world study in patients with RA, where filgotinib proved to be the only JAKi with a neutral effect on the LDL/HDL-c ratio (Benucci et al., 2024).

These results may be clinically relevant because LDL-c is an established atherogenic component of the lipid profile, and the main guidelines on cardiovascular risk prevention recommend reaching specific LDL-c targets, considering other lipid parameters less critical (Mach et al., 2019).

However, even in the long-term safety follow-up of tofacitinib, cholesterol increase has not been clinically meaningful since the Reynolds cardiovascular risk score (which assesses the risk of cardiovascular events at 10 years) did not increase after 8 weeks of treatment, and only 4.8% of patients had to start a lipid-lowering therapy during the study (Sands et al., 2020).

It should be mentioned that in IBD blood lipid levels are typically lower than in the general population and inversely related to disease activity; therefore, a treatment capable of reducing disease activity is expected to increase blood cholesterol levels (Charles-Schoeman et al., 2015; Chen et al., 2023). It has been hypothesized that the effect of JAKi on cholesterol levels is not direct but due to drug-related improvement of inflammation. This has been confirmed by a study on tofacitinib, where in treated patients showed a reduction in the activity of the cholesterol-esterase enzyme was observed, which correlated with the inflammation burden reduction (Charles-Schoeman et al., 2015).

### 3.4 Feasibility of co-treatment with JAKis and statins

Although rarely needed both in clinical trials (<5% of patients) and clinical practice, statin therapy initiation may be required to reduce the JAKis-related LDL-c elevation (Sands et al., 2020).

In patients treated with JAKis, statin therapy can effectively restore normal LDL-c levels (European Medicine Agency, 2024d; European Medicine Agency, 2024b; European Medicine Agency, 2024c; European Medicine Agency, 2024a). However, the Summaries of Product Characteristics (SmPCs) of different JAKis not always include comprehensive information about JAKi-statins interactions (Table 4). For example, tofacitinib SmPC do not mention possible interactions with statins, while baricitinib SmPC only excludes the risk of interactions with simvastatin.

**TABLE 4:** JAKis impact on statins pharmacokinetics according to their respective SmPCs.

	Tofacitinib	Baricitinib	Upadacitinib	Filgotinib
Simvastatin	NM	No clinically meaningful changes in the PK.	NM	NM
Rosuvastatin	NM	NM	33% AUC reduction No dose adjustment required	42% AUC increase No dose adjustment required
Atorvastatin	NM	NM	23% AUC reduction No dose adjustment required	No dose adjustment required
Pravastatin	NM	NM	NM	No dose adjustment required

SmPC: summaries of product characteristics, NM: not mentioned in SmPC, PK: pharmacokinetics, AUC: area under the curve, JAKi: Janus kinase inhibitors. Adapted from European Medicine Agency (2024d), European Medicine Agency (2024b), European Medicine Agency (2024c), European Medicine Agency (2024a).

Filgotinib and upadacitinib are the only JAKis with studies assessing the pharmacokinetic interactions with statins in healthy volunteers (Anderson et al., 2022; Mohamed et al., 2021).

The filgotinib study was conducted to clarify whether the *in vitro* inhibitory effect of this JAKi on OATP-1B1 and OATP-1B3 (which are responsible for statin transport) could have pharmacokinetic consequences in humans. Notably, in the 2020 version of the filgotinib SmPC, co-administration of this JAKi and statins was contraindicated. The study showed that filgotinib had no clinically relevant effects on atorvastatin, pravastatin, or rosuvastatin exposure and, as a result, the contraindication to co-administration was removed from SmPC (Anderson et al., 2022; European Medicine Agency, 2024a).

The upadacitinib study gave similar results, showing that the JAKi had no clinically relevant effect on rosuvastatin and atorvastatin pharmacokinetics; as a result, the current upadacitinib SmPC reports that no dose adjustments are necessary when co-administered with these statins (Mohamed et al., 2021; European Medicine Agency, 2024c).

## 4 JAKi and male fertility

All JAKis, except for upadacitinib, have shown a potential impact on fertility in animal models (Table 5). While with tofacitinib and baricitinib a possible reduction in female fertility emerged in animal models, filgotinib showed a reduction in male fertility. Indeed, in the first version of this JAKi SmPC included a warning about the possible treatment-associated impairment of spermatogenesis and histopathological effects on male reproductive organs.

**TABLE 5:** JAKis impact on male fertility according to their respective SmPCs.

JAKi	Fertility information from SmPC
Tofacitinib	Formal studies of the potential effect on human fertility have not been conducted. Tofacitinib impaired female fertility but not male fertility in rats
Baricitinib	Studies in animals suggest that treatment with baricitinib has the potential to decrease female fertility while on treatment, but there was no effect on male spermatogenesis
Upadacitinib	The effect of upadacitinib on human fertility has not been evaluated. Animal studies do not indicate effects with respect to fertility
Filgotinib	In animal studies, decreased fertility, impaired spermatogenesis, and histopathological effects on male reproductive organs were observed The data from two dedicated Phase 2 clinical studies (MANTA and MANTA RAy, n = 240) to evaluate the human testicular safety in men with inflammatory arthritis diseases and inflammatory bowel diseases did not reveal a difference between treatment groups in the proportion of patients who had a 50% or more decrease from baseline in semen parameters at week 13 (pooled primary endpoint: filgotinib 6.7%, placebo 8.3%) and at week 26 Further, the data did not show any relevant changes in sex hormone levels or change from baseline in semen parameters across treatment groups. Overall, these clinical data were not suggestive of filgotinib-related effects on testicular function Animal studies did not indicate effects with respect to fertility in females

SmPC: summaries of product characteristics, JAKi: Janus kinase inhibitors. Adapted from European Medicine Agency (2024d), European Medicine Agency (2024b), European Medicine Agency (2024c), European Medicine Agency (2024a).

Following these preclinical observations, in 2020, the FDA requested Gilead Science to conduct a randomized, controlled trial in humans to elucidate the effects of filgotinib on male fertility.

Therefore, the MANTA and MANTA-RAy studies were designed and conducted to collect long-term data on the effects of filgotinib on testicular function and sperm parameters and to evaluate the reversibility of these effects after drug discontinuation (Reinisch et al., 2023).

MANTA and MANTA-RAy were two international, randomized, placebo-controlled studies that had enrolled, respectively, patients with IBD and patients with autoimmune rheumatic diseases (RA, PsA, axSpA or non-radiographic axSpA).

The primary endpoint of both studies was the proportion of participants with ≥50% reductions from baseline in sperm concentration at week 13. Secondary endpoints were several other male fertility parameters, such as sperm motility, sperm count, sperm concentration, ejaculate volume, and sperm morphology. The evaluation of the reversibility of filgotinib effects on male fertility was an exploratory endpoint of the studies.

Both studies included an OLE in which patients could continue filgotinib for up to 195 (MANTA) or 156 (MANTA-RAy) weeks.

More than 600 patients were screened, and 248 were randomized: 123 received filgotinib 200 mg day, and 125 received equivalent placebo. The majority of patients completed the double-blind phase of the studies.

The patient population at baseline consisted predominantly of young adults, typically Caucasian (70%); the majority of patients had UC (50.8%) or axSpA (25.8%). Enrolled IBD patients had moderately active disease.

The pooled analysis of MANTA and MANTA-Ray data showed similar and low proportions of patients treated with filgotinib versus placebo meeting the primary endpoint of ≥50% reduction from baseline in sperm concentration at week 13 (6.7% vs. 8.3% respectively; difference −1.7% [95% CI -9.3%–5.8%]).

Similarly, no statistically significant differences between the filgotinib and placebo arms in any of the secondary endpoints were detected.

In 16 of the 18 patients whose sperm parameters had changed significantly after filgotinib discontinuation, the values returned to baseline levels, demonstrating the reversibility of filgotinib effect.

The authors concluded that the results observed in animal models were not confirmed in humans, where filgotinib shows no measurable effect on sperm parameters and other indicators of male fertility.

These results led to the modification of filgotinib SmPC, with the removal of the male fertility warning. Notably, filgotinib is nowadays the only JAKi with evidence from an RCT supporting its lack of impact on male fertility.

## 5 Discussion

The evidence analyzed in this paper confirms the presence of clinically relevant differences between JAKis and calls into question their interchangeability. In spite of these recognized differences there are no clear guidelines to help clinicians make the choice, and there are many potentially relevant and yet unexplored factors to consider when selecting the most appropriate JAKi.

For example, a particularly intriguing topic is the selection of the most appropriate JAKi based on the disease stage. Since cytokine expression patterns progressively change during the course of IMIDs, different JAKis may be more or less effective at different time points due to different selectivity for cytokine pathways. However, available data are still inadequate in suggesting strategies for using JAKi sequentially in IMIDs.

Moreover, no reliable biomarkers are available today to guide JAKi selection. Unlike biologics, the search for biomarkers for JAKi is still in its infancy. Several biomarkers have shown promising results in recent studies, but their use in clinical practice is not yet recommended (Cuccia et al., 2024; Roblin et al., 2022; Tchetina et al., 2022; Benucci et al., 2022).

On the basis of what has been illustrated in this paper, however, it is already possible to identify the clinical contexts in which the choice of filgotinib may be appropriate, given the peculiar characteristics of this JAKi.

For example, filgotinib may be particularly suitable for poly-treated patients using drugs with hepatic metabolism, as it is the only JAKis metabolised in the intestine by CES2. This is the reason why filgotinib may limit the risk of adverse events or reduced efficacy resulting from pharmacokinetic interactions with other drugs. This aspect is crucial in patients with IMIDs: it has been estimated that 40% of patients with RA and 60% of those with IBD have ≥2 concomitant diseases, which makes the management of polytherapy a very common need of rheumatologists and gastroenterologists (Gunderson et al., 2021; Argollo et al., 2019). In the context of polytherapy, it is also important to underline that filgotinib and upadacitinib are the only JAKis with a proven neutral pharmacokinetic effect on statins in humans (Anderson et al., 2022; Mohamed et al., 2021).

In addition, filgotinib may be an appropriate choice for male patients with UC who plan to have children. This is a relevant subset of patients with UC since this disease is more prevalent in males and its onset typically occurs between the ages of 30 and 40 (da Silva et al., 2014). The safety of filgotinib use in these subjects is supported by two studies (MANTA and MANTA-Ray) that have demonstrated its neutral effect on sperm parameters (Reinisch et al., 2023).

Finally, in the absence of head-to-head studies, indirect comparison of filgotinib data with other JAKis (particularly tofacitinib) suggests that this drug may have a more favourable benefit/risk ratio. This is a direct consequence of filgotinib preferential selectivity for JAK1, whose inhibition is associated with JAKis anti-inflammatory activity. The inhibitory effects of filgotinib on JAK2 and JAK3, on the other hand, are negligible, and this is the reason for this drug’s lower propensity to cause the adverse events commonly associated with JAKis.

In conclusion, JAKis represent a valuable therapeutic option in IMIDs treatment, but they are not a homogeneous class of drugs. There are major differences in the pharmacokinetic and pharmacodynamic profiles of these molecules. It is essential to be aware of these differences in order to choose the most appropriate JAKi for each patient. In this article, we have reviewed the most relevant evidence on JAKis to help differentiate the characteristics of individual molecules. We have summarized the results of our work in seven points to consider that can support rheumatologists, gastroenterologists and dermatologists in choosing a JAKi for their patients (Table 6).

**TABLE 6:** Points to consider when selecting a JAKi for patients with IMIDs.

Points to consider	
1	JAKis are characterized by different mechanisms of action in terms of binding selectivity for JAK isoforms. JAK1 and TYK2 have a prevalent effect on inflammatory signal transduction. In addition to their involvement in inflammatory signals, JAK2 and JAK3 regulate hematopoietic processes. Filgotinib preferentially inhibits JAK1
2	JAKis are characterized by different mechanisms of biotransformation: baricitinib, tofacitinib and upadacitinib are metabolized in the liver by the P450 cytochrome system. Filgotinib is entirely metabolized in the intestine by different enzymatic sets (CES2). This may benefit poly-treated patients using drugs with predominant hepatic metabolism
3	In humans, filgotinib and upadacitinib have not demonstrated pharmacokinetic interactions with atorvastatin, pravastatin, and rosuvastatin. Therefore, it is conceivable that co-treatment with these JAKis and these statins does not result in clinically relevant effects
4	The use of filgotinib 200 mg/day, according to its label, has no impact on sperm parameters in patients with IMIDs
5	From a safety perspective, the use of JAKi in patients with RA and risk factors has been associated with adverse cardiovascular events. However, there are no data describing the risk in UC patients or comparing individual molecules
6	There are no markers that can guide the selection of specific JAKis to be used in specific types of patients or at a particular stage of the disease

IMID: immune mediated inflammatory disease, JAKi: Janus kinase inhibitor, JAK: janus kinase, CES2: carboxyl-esterase 2, RA: rheumatoid arthritis, UC: ulcerative colitis.
